# Comparison of Luting Cement Solubility: A Narrative Review

**DOI:** 10.3390/dj12110365

**Published:** 2024-11-15

**Authors:** Deok Yong Kim, Nona Aryan, Nathaniel C. Lawson, Kyounga Cheon

**Affiliations:** 1 Department of Pediatric Dentistry, School of Dentistry, University of Alabama, Birmingham, AL 35294, USA; dkim5@uab.edu (D.Y.K.); na7@uab.edu (N.A.); 2 Division of Biomaterials, School of Dentistry, University of Alabama, Birmingham, AL 35294, USA; nlawson@uab.edu

**Keywords:** dental luting cements, glass-ionomer cement, resin-modified glass ionomer cement, resin cement

## Abstract

**Background:** Dental restoration success relies on the physical properties of luting cements. Luting cements fill the space between teeth and the restoration, provide retention and protection from occlusal forces, and act as a barrier to microleakages in the oral environment. **Objective:** This review aims to evaluate and compare the solubility of the three most used dental luting cements: glass ionomer (GI), resin-modified glass ionomer (RMGI), and resin cement (RC). **Methods:** The studies selected for review compared the solubilities of combinations of GI, RMGI, and RC in solutions with different pH levels to replicate acidic oral pH. **Results:** A review of the studies concluded that resin cement had the overall lowest degree of solubility at all pH values and all storage periods, followed by RMGI and GI cement. **Conclusions:** The success of the restoration is dependent upon the choice of luting cement. The results of the studies reviewed show that all dental luting cements showed some degree of dissolution. Resin cement overall demonstrated the least amount of solubility, followed by RMGI and GI cement.

## 1. Introduction

The success of restorations in terms of retention is dependent on the physical properties of the luting cements or dental cement. Solubility is one of the most critical factors when assessing the effectiveness and quality of luting cements in restorative dentistry [1]. Luting cements fill the space between the tooth and restoration and protect teeth from the harmful effects of occlusal force as well as serving as a barrier against leakages. Dental luting cements at the margin of the restoration are in constant contact with the oral flow and are subject to dissolution. Therefore, the ideal dental luting cements will be resistant to disintegration and dissolution and avoid creating an environment that is susceptible to plaque and bacterial accumulation, secondary caries, and the debonding of the restoration [1].

In pediatric dentistry, stainless steel crowns (SSCs) are frequently utilized to restore primary teeth or permanent teeth, which require the use of dental luting cement. SSCs have high clinical success records in terms of retention. However, one of the critical failures in SSC restorations is caused by cementation-associated problems, including problems regarding the choice of materials and techniques [1]. Conventionally, an SSC is cemented using zinc phosphate, GI, RMGI, or resin-based cement. Studies have determined that GI cement is superior to RMGI cement, which is followed by resin cement in terms of potential demineralization inhibition. However, RMGI demonstrated an equally successful clinical outcome to GI cements in terms of bond strength (*p* < 0.124) [1,2]. This narrative review summarizes and compares the solubilities of GI, RMGI, and resin cements at various pH values and storage periods. This review is unique in that it aims to provide a comprehensive summary of various brands within a specific category of cement type. Moreover, this review provides solubility results for different ranges of pH values and a wide range of storage periods. The purpose of this review is to evaluate the relative solubility of frequently used dental luting cements and identify the type with the lowest solubility for routine use in dental practice.

## 2. Materials and Methods

### 2.1. Selection Criteria

In vitro studies that compared the solubility of any combination of RC, RMGI, and GI in artificial saliva were included in this review. All studies included prepared the luting agent samples on a disk and recorded their weight prior to their submersion in solution. After the submersion period, a drying cycle was carried out prior to the recording of the weight again. Studies with a storage period of less than 7 days were excluded from the review.

### 2.2. Search

Articles were searched in PubMed database from 1960 to 2024 with the search phrases “Solubility of dental cement”, “Solubility of dental luting cement”, “Solubility of dental luting cement”. In the advanced setting, the search terms “resin cement solubility”, “glass ionomer solubility” and “resin modified glass ionomer solubility” were added.

### 2.3. Zinc Phosphate Cement

**Figure 1:**
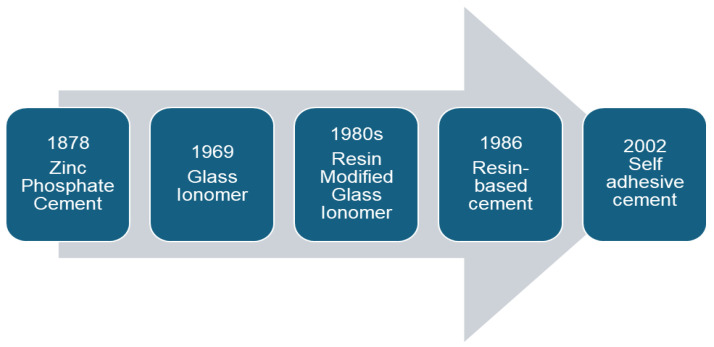
History of the development of dental luting cement [8,9,10].

As shown in Figure 1, zinc phosphate cement (ZPC) was first used in 1878 as the “gold standard” for the cementation of restorations with years of clinical success [3,4,5,6,7]. The strengths of zinc phosphate cement include a high compressive strength of up to 104 megapascals and a working time of about 45 s [4]. This cement is used to lute metal and metal–ceramic full-coverage crowns and fixed partial dentures [3,4,5]. However, ZPC demonstrated the disadvantages of high solubility in saliva, a low tensile strength, and the potential for hypersensitivity due to a low pH at the time of cementation [4].

### 2.4. Glass Ionomer Cement

Glass ionomer (GI) cement was formulated in 1969 by Wilson and Kent [11] and became the most frequently used, definitive luting cement by the 1990s. The advantages of GI cement include its ease of mixing, flow, ability to adhere to tooth and base metals, ability to release fluoride, transparency, high strength, and relatively low cost. GI cement also has a lower propensity to change size with a low thermal expansion coefficient. GI cement comes in a powder–liquid form and its physical properties can vary depending on the powder-to-liquid mixing ratio; therefore, following the manufacturer’s recommendation is highly critical. There are encapsulated cements available that contain consistent ratios that can eliminate this potential variability and difficulty of use and ensure accurate, recommended proportions. GIC also has a low pH that can cause hypersensitivity after cementation [3,4,5,6].

### 2.5. Resin Modified Glass Ionomer

RMGI is a hybrid material introduced in the 1980s and is made by adding water-soluble polymerizable resin components to conventional GI cement. The advantages of RMGI include a high fracture resistance, favorable physical and mechanical properties, strong bonding to enamel and dentin, and a higher resistance to wear. RMGI cements were made in an attempt to overcome the weaknesses of conventional GI cements, such as their relatively lower strength and high solubility. This material is easy to use, exhibits less film thickness, and has favorable esthetic properties. RMGI cement was shown to have superior physical and mechanical properties compared to conventional GI cement [7].

### 2.6. Resin Cement

Resin cement is the most recently developed dental cement. Unlike other luting cements, derived from powder and a liquid mixture to form a hydrogel, resin cement forms a polymer matrix to fill and seal the gap between the tooth and the restoration [11]. The first resin cements experienced failures due to a high degree of polymerization shrinkage and inadequate enamel and dentin bonding. Modern resin cements are more predictable and adaptable, allowing for them to be used in many different cases [11]. The use of resin cement is ideal in many cases due to its versatility, low thermal expansion, high compressive and tensile strengths, and ideal esthetic qualities. However, there are also downfalls such as difficulties removing excess cement and the sensitivity of the technique [11]. In delivering metal and metal–ceramic restorations, resin-luting cement, such as Rely X Unicem, is as effective in terms of performance as conventional ZPC [12]. Resin cement can either be adhesive or self-adhesive [12]. Adhesive cement requires the tooth to be acid-etched with phosphoric acid prior to the application of adhesive components [13]. Self-adhesive cement does not require acid-etching and adhesive application [13]. At this time, long-term clinical data are not sufficient to support the routine use of resin cement over conventional luting cement [11].

## 3. Results

### 3.1. Retention from Luting Cement

An in vitro study using 55 extracted primary first molars was conducted to compare the retentive ability of four luting cements in cementing SSC [14]. The luting cements included in the experiment were resin, GI, zinc phosphate, and polycarboxylate cement. The success of restoration depends on the selection of an appropriate luting cement, considering its mechanical properties. By filling in the empty space between the tooth and restoration, luting cements provide retention and adhesion. Polycarboxylate cement showed maximum retentive strength and zinc phosphates demonstrated the lowest retentive strength in this study. This study found that there was no significant difference between resin cement and GI cement concerning their ability to be retentive [14]. As retentive strength was found to be comparable between GI and resin cement, and considering that SSCs face constant exposure to the oral environment and dynamic oral fluids, it is strongly advised to examine how solubility will affect the success of SSC cementation.

Furthermore, the retention of the luting cement used to cement permanent crowns was examined in a review of crown pull-off tests. A total of 18 studies that performed pull-off tests on extracted teeth were reviewed. The cements compared were zinc phosphate, GI (Ketac-Cem), and resin cement (Panavia and RelyX Unicem). The studies used extracted molars and premolars and prepared them for either metal alloy crowns (16 studies) or ceramic crowns (2 studies). Resin-based cement demonstrated higher stress failure compared to GI, with an average percentage difference of 32.2% (*p* = 0.03), and GI had higher stress failure compared to zinc phosphate cement, with average percentage difference of 25.1% (*p* = 0.02). Resin cement demonstrated superior retention during the pull-off test; however, previous studies have declared that different properties of cement, including shrinkage, expansion, water uptake, and water solubility, can heavily affect the success of cementation [15].

### 3.2. Solubility of RMGI vs. Resin Cement

A study performed by Gavranović-Glamoč et al. compared RMGI (GC Fuji Plus) and two resin cements (Multilink Automix and Variolink II). Samples were prepared according to ISO standard 4049:2009, which defines the specific requirements for dental polymer-based restorative materials [13]. Teflon molds are used to shape luting cement into disk shapes. RMGI specimens were prepared with a polyester film and metal film. The second metal plate was placed on top to eliminate surplus material, and metal plates were held together by clamps and immediately placed in a sealed environment, before being kept at 37 ± 1 °C for 60 min. All samples were then refined and polished with ultra-fine silicon carbide paper until a uniform diameter was obtained. For dual cure resins, metal plates were replaced by glass plates to polymerize specimens. After materials were formed into disks, all samples were then stored in desiccators with silicate gel and stored in an incubator for 22 h. After 22 h, samples were placed together in another desiccator that was kept at a stable temperature of 23 ± 1 °C for two hours, then weighed at an analytical balance until a constant mass was obtained.

The solubility of the luting cement in question was compared with three different pH values. Solution 1 was distilled water, solution 2 had a pH value of 7.4 to reflect slightly basic artificial saliva, and finally, solution 3 had a pH value of 3.0 to reflect an acidic environment. Samples were then submerged in solutions prepared for each testing condition and solubility was measured at five time periods of 24 h, 48 h, 72 h, 96 h, and 168 h. Samples were taken out of the solution, rinsed out with water, air-dried for 15 s, and weighed sixty seconds after being taken out of the corresponding storage solution to record the measured mass. The formula used to calculate solubility (Wsl) was Wsl = (m1 − m3)/V, where m1 is the mass of specimens before submersion, m3 is the mass of samples after drying, and V represents the volume of specimens [13]. The results are summarized in Table 1.

When the solubility of GC Fuji Plus (RMGI) was compared to resin cement, Multilink Automix, and Variolink II in solutions with pH 7.4 and pH 3.0, GC Fuji plus showed statistically significant higher solubility in comparison with the Variolink II and Multilink Automix in all solutions, except in the solution of pH 7.4, where no statistically significant difference in solubility was confirmed between Multilink and GC Fuji Plus. In the acidic (pH 3.0) solution, Multilink showed higher solubility compared to Variolink II, with a *p*-value < 0.016, and GC Fuji Plus showed significantly higher solubility values in the pH 3.0 solution (*p* < 0.009) [13]. In the acidic solution, resin cement showed significantly lower solubility compared to RMGI. Additionally, a difference in solubility between Multilink and Variolink was observed, suggesting that even within the same type of cement (i.e., resin), possible significant differences in solubilities exist.

### 3.3. Solubility of Resin vs. RMGI vs. GI

In another in vitro study conducted by Mehta et al. [16], a total of eight luting cements were compared. Interestingly, this study compared both permanent dental luting cement and temporary luting cement to determined whether certain cements are more appropriate in situations where temporary cement is needed [16]. The permanent cements that were compared were Rely X lute 2, Zinc phosphate, zinc polycarboxylate, Rely X U-200, and GI cement G.C (Fuji). The temporary cements that were compared were zinc oxide eugenol (ZOE), Oratemp NE, and Temposil. Each material was prepared at an equal size (20 mm by 1.5 mm) using a similar method as the above studies, and the manufacturer’s recommendations were followed when mixing. Samples were then submerged in solutions of varying pH values (3, 5, 7, or 9) and weighed to test for dissolution at 24 h, 72 h, 7 days, and 28 days. The results are shown in Table 2. A comparison within the temporary cement groups demonstrated that Temposil had the lowest solubility after 28 days and ZOE showed the greatest solubility. The study of Temposil at differing pH values showed that solubility was the highest after 28 days in pH 3 and the lowest in distilled water. For Oratemp NE and ZOE, solubility was highest in the pH 3 solution and lowest in the pH 9 solution. In the permanent cement group, Rely X U-200 had the lowest solubility at 28 days, followed by Rely X lute-2, RMGI, Zinc polycarboxylate, and zinc phosphate, which demonstrated the highest solubility among the permanent cement materials. Interestingly, for GI cement, solubility was highest at pH 3.0 and lowest at pH 9, while for Rely X U-200, solubility was highest at pH 5 and lowest at pH 9. Zinc phosphate and polycarboxylate cements showed similar solubility results, with the highest solubility at pH 3 and the lowest in distilled water [16]. These results were intriguing in that, between the same type of material, the highest and lowest solubility results were different at different pH levels. Overall, a similar pattern was observed in that resin cements had the lowest solubility, followed by RMGI and GI, at all pH values.

### 3.4. Solubility of Resin vs. GI

Yoshida et al. have stated that one of the most crucial properties of luting materials that must be considered is their ability to resist dissolution and disintegration. To represent the conditions of the oral cavity as closely as possible in vitro, pH values of 5.7 and a lactic acid solution of pH 4.0 were tested for comparison. The materials tested were resin (Panavia 21, all-bond C&B, super-bond) and GI cement Fuji I. the results are presented in Table 3. It should be noted that Panavia and all-bond both had filler components, and the super-bond did not contain fillers. The results of the experiment reinforced that resin cement had the lowest solubility compared to GI cement and that all cement types showed markedly increased solubility in an acidic environment of pH 4.0 [17,18,19]. This result further supports the findings of previous studies, showing that resin cements have the lowest solubility.

### 3.5. Oral Salivary pH Change After Consumption of Soft Drinks in Children

Sanchez and Preliasco studied the salivary pH changes of children after the consumption of soft drinks [19]. pH changes after the consumption of different soft drinks, including Coca-Cola, Sprite, Ades N, and chocolate milk, were examined and the results indicated that pH values showed a statistically significant drop. It was also demonstrated that the reduction in pH value was maintained between 5.5 and 6.2 [19]. Another study on forty-five 12-year-old children from public schools in Itatiba, Brazil, measured salivary pH following the consumption of acidic beverages. The authors explained that, upon contact with saliva, the acid in the beverage releases hydrogen ions and results in a decrease in salivary pH. Immediately after intake, pH reduced to 6.26 and slowly increased over 15 min. At 15 min after the consumption of an acidic soft drink, salivary pH was 6.64 on average, and the results were statistically significant [20]. These results demonstrate that salivary pH is significantly affected by the beverages we consume and this reduction in pH lasts for at least 15 min in children. It is important to consider these results, as numerous studies have found that a large percentage of young children consume sugary soft drinks daily [21]. According to these studies, significantly higher cement solubility was observed in acidic conditions. Considering the salivary pH decrease upon the consumption of soft drinks, the association between pH and decreases and increases in dental cement solubility should be further evaluated.

## 4. Discussion

From the studies and experiments reviewed in this article, consistent overall results were observed across the board. Resin cement showed the highest resistance to dissolution and disintegration, followed by RMGI cement and GI cement. All samples were prepared similarly, in the shape of a disk of about 15–20 mm diameter with a thickness of approximately 1.5 mm [13,16,17,18]. While studies were conducted using a varying range of pH values and sample immersion periods, the overall trend was similar in that all luting cements showed increased solubility in more acidic conditions and with increased storage periods (Table 4).

The significantly higher solubility reported for RMGI cement in an acidic solution compared to resin cement may be attributed to the hydrophilic nature of RMGI cement. RMGI cement showed a significantly higher solubility than resin cement [13]. However, through the introduction of a resin matrix in its composition, RMGI cement can limit the diffusion of the solvent into the cement, and therefore exhibits less solubility compared to conventional GI cement [17]. The results of these studies are essential to the field of dentistry in that the solubility of cement is one of the most essential properties determining the success of dental restorations. While testing the solubility of dental cements in human oral cavities poses a challenge, the studies made attempts to closely mimic the conditions of the oral cavity by controlling natural salivary pH and acidic salivary pH.

Yoshida et al. showed a change in the pH of distilled water (original pH 5.7) and lactic acid solution after 30 days of sample suspension [17]. According to the data, distilled water and an acidic solution in which resin cements were suspended showed no change in pH over 30 days. However, distilled water that contained conventional luting cements showed an increase in pH to nearly 7.0 at the end of the 30 days. This result occurred due to intermediates being formed during the dissolution of the luting cement material. More specifically, zinc and magnesium are released from zinc phosphate and polycarboxylate cement, and aluminum and silicon are released from GI cement. This explains the increase in the pH of the two solutions that contained the conventional luting cement. However, resin cement demonstrated no change in pH as there was minimal release of methacrylate monomers into the solution. This additionally goes to show that resin cements experience markedly less solubility in both distilled water and lactic acid solutions. When solubility data are collected and the relationship between solubility and immersion period is evaluated through regression analysis, a statistically significant positive correlation is shown. This linear relationship can help to estimate solubilities over an extended period. The authors predicted that the three conventional luting cements will disintegrate within two years. On the other hand, resin cements are expected to completely break down between 3 and 9 years for super-bond and all-bond, whereas Panavia 21 is estimated to take 35 years to dissolve by 5%. This result is interesting and leads to questions regarding the role of fillers and the composition of resin cement and what component could affect the rate of dissolution to this extent [17].

The strength of this review lies in its comparing different types of cement brands. While resin cements overall had the lowest solubility compared to RMGI and GI cements, their solubility was different depending on the specific brand of resin cement used. For example, within the self-adhesive resin cements, Maxem Elite had highest solubility value at an acidic pH (1.11) and Rely X Unicem had the lowest solubility (0.13), as shown in Table 4. These results can aid providers in choosing one type of resin cement versus another. This review has potential limitations. The data collected from previous studies were not obtained in identical settings and conditions. The results were obtained under laboratory conditions with a relatively short observation period considering the length of the restoration life in the human mouth. Additionally, dental materials are evolving fast. Future studies should focus on evaluating the solubility of new dental luting agents for a longer period of time under conditions that better reflect the natural oral environment.

## 5. Conclusions

Reiterating the fact that the success of restorations is dependent upon the luting cements used, these results apply not only to permanent teeth but also to primary teeth that are restored by stainless steel crowns and strip crowns. Considering the large percentage of children that consume sugary carbonated beverages, which were shown to lower salivary pH for up to 15 min after consumption, the solubility of dental cementing materials that come into contact with such beverages should also be examined. When selecting a luting cement for cement restorations, clinicians need to consider all aspects of the luting cement, including its physical and mechanical properties, biocompatibility, water sorption, and solubility.

In this review, we concluded the following:

- Overall, resin cements demonstrated the lowest solubility across all pH values and storage periods.
- The water solubility of dental cement was shown to have a critical impact on restoration success.
- Therefore, future studies should focus on improving the replication of dynamic oral cavity environments to test dental cement materials in different solutions, such as following the consumption of carbonated beverages and sports drinks, which are frequently consumed by the public, using shorter intervals to determine the solubility of different dental luting cements.

## Figures and Tables

**Table 1:** Solubility of resin vs. RMGI. Finding: RMGI cement exhibited statistically significantly higher solubility compared to resin cements in distilled water and at an acidic pH (*p* < 0.009) and no significant difference was observed in a pH 7.4 solution between RMGI and Multilink cement (*p* = 0.024).

Reference	Type of Cement	Name of Cement	Solubility in Distilled Water	Solubility in Acidic pH	Solubility in Basic pH
[13]	Resin	Multilink Automix	−3.06	−4.10	−3.20
		Variolink II	−5.25	−5.41	−5.29
	RMGI	GC Fuji PLUS	7.12	13.22	3.46

**Table 2:** Solubility of resin vs. RMGI vs. GI. Findings: Rely X U-200 resin cement demonstrated the lowest solubility at the end of the 28-day test period, followed by Rely X Lute 2 (RMGI), and lastly, GC GIC (final weight—initial weight).

Reference	Type of Cement	Name of Cement	Solubility in Distilled Water	Solubility in Acidic pH	Solubility in Basic pH
[16]	Resin	Rely X U200	0.008	0.012	0.008
	RMGI	Rely X Lute 2	0.020	0.020	0.020
	GI	GC GIC	0.040	0.044	0.038

**Table 3:** Solubility of resin vs. GI. Finding: All resin cements showed lower solubility compared to GI cement and all cement types showed the highest solubility in acidic solution.

Reference	Type of Cement	Name of Cement	Solubility in Distilled Water	Solubility in Acidic pH
[17]	Resin	Panavia 21	0.89	1.36
		All bond C&B	0.23	0.34
		Super-Bond	1.10	1.34
	GI	Fuji I	2.65	3.32

**Table 4:** Solubility of conventional resin vs. self-adhesive resin vs. RMGI.

Reference	Type of Cement	Name of Cement	Solubility in Acidic pH
[18]	Conventional Resin	Panavia F (PF)	0.67
		Rely X ARC (RA)	0.46
	Self-adhesive Resin	Rely X Unicem (RU)	0.13
		Breez (BZ)	0.93
		Maxcem Elite (MX)	1.11
		BisCem (BC)	0.94
	RMGI	FujiCem (FC)	4.83
		Rely X Luting Plus (RL)	3.25
		Fuji Plus (FP)	1.99

## Data Availability

The original contributions presented in the study are included in the article; further inquiries can be directed to the corresponding authors.
